# The Prevention of Common Diseases in Childhood

**Published:** 1913-03-15

**Authors:** 


### BIBLIOGRAPHY.

The Prevention of Common Diseases in Childhood. This is a little book of about one hundred pages on the subject of mouth hygiene by Dr. J. Sim Wallace of London, England, and printed by Bailliere, Lindall and Cox, 8 Henrietta St., London. It can be had through American booksellers.

The author in this collection of essays which he has contributed to various medical and dental societies is endeavoring to show that dental caries is a preventable disease, largely by the method of a regulated diet. He also sets forth with considerable force that disease of the teeth and oral cavity are most important factors in introducing through the mouth and *primae viae* systemic infections that are difficult to eradicate and wholly unnecessary. He crticises the medical professions for overlooking this important source of disease while centering its attention almost entirely on efforts to determine food values with which the malnutrition of the body may be corrected. Few children suffer from lack of nourishing food, but too many suffer the results of unclean and unsanitary food because of its lack of proper preparation for digestion in the mouth and its contamination with infectious food debris and putrid oral contents. Dr. Wallace has done a good work and this little book ought to be read by every dentist, physician and mother in our country.

